# Budd-Chiari syndrome in a 33-year-old woman with hypercoagulable state: A case report

**DOI:** 10.1016/j.radcr.2025.03.023

**Published:** 2025-04-12

**Authors:** Laleh Abbasi, Alireza Motamedi, Ali Kiaee, Fatemeh Abbasi, Ommolbanin Younesian, Nazgol Khodaie

**Affiliations:** a School of Medicine, Tonekabon branch, Islamic Azad University, Tonekabon, Iran; b Tabriz University of Medical Sciences, Tabriz, Iran

**Keywords:** Budd-Chiari syndrome, Hepatic venous outflow obstruction, Hypercoagulable

## Abstract

Budd-Chiari syndrome (BCS) is a rare disorder caused by hepatic venous outflow obstruction, often linked to underlying prothrombotic conditions. This case describes a 33-year-old woman who presented with abdominal pain and ascites and was diagnosed with BCS secondary to deficiencies in protein C, protein S, and antithrombin III. She also had a history of epilepsy, bipolar disorder, and poor medication adherence. Imaging studies, including Doppler ultrasound and contrast-enhanced CT, played a crucial role in confirming the diagnosis. Despite treatment with anticoagulation and diuretics, recurrent ascites and behavioral issues complicated management, leading to a poor prognosis. This case highlights the importance of early recognition, imaging in diagnosis, and evaluation of prothrombotic disorders in patients with BCS to improve outcomes.

## Introduction

Budd-Chiari syndrome (BCS) is a rare condition caused by hepatic venous outflow obstruction, excluding cases due to cardiac, pericardial, or veno-occlusive disease [1]. It is classified into primary BCS, resulting from a venous process such as thrombosis or phlebitis, and secondary BCS, which occurs due to external compression or invasion of the hepatic vein or inferior vena cava (IVC), often by malignancy [2,3]. BCS can also be categorized based on disease duration and severity into acute fulminant, acute nonfulminant, subacute, and chronic forms [2]. Common symptoms include right upper quadrant pain, hepatomegaly, ascites, esophageal variceal hemorrhage, jaundice, coagulopathy, and encephalopathy [1]. The prevalence is approximately 1 in 100,000, with an underlying disorder identified in 80% of cases, most commonly myeloproliferative diseases, acquired or inherited thrombophilia, and other hypercoagulable states [2,4]. Imaging plays a crucial role in diagnosing BCS, with Doppler ultrasound being the initial modality, while contrast-enhanced CT or MRI provides detailed vascular assessment [2]. This report presents a 33-year-old woman who developed BCS due to protein C, protein S, and antithrombin III deficiencies. The case highlights the importance of thorough evaluation for prothrombotic conditions and the critical role of imaging in early diagnosis and management.

### Case presentation

A 33-year-old woman presented to the emergency department with progressive abdominal pain and distension for 2 weeks. Her symptoms began with intermittent, generalized abdominal pain, which acutely worsened and localized to the right upper quadrant with radiation to the right shoulder, accompanied by nausea and shortness of breath. Examination revealed massive ascites, diffuse abdominal tenderness (more pronounced in the epigastric region), and bilateral pitting edema. She was hemodynamically stable with no jaundice or overt signs of portal hypertension.

Her medical history was significant for epilepsy, managed with sodium valproate, and bipolar disorder, for which she had been previously hospitalized but had discontinued prescribed medications. She also had a history of substance use, including methamphetamine, alcohol, and cigarettes. There was no prior history of thrombotic events, hematologic disorders, or autoimmune diseases.

Doppler ultrasound revealed collapsed suprahepatic veins, nonvisualization of the hepatic vein-inferior vena cava junction, and abnormal hepatic vein flow, suggestive of Budd-Chiari syndrome (BCS). Contrast-enhanced CT confirmed the diagnosis, demonstrating an enlarged, nodular liver with caudate lobe hypertrophy, nonvisualized suprahepatic veins, narrowing of the hepatic segment of the inferior vena cava, and prominent collateral vessels. MRI with venography further delineated hepatic vein thrombosis.

Laboratory tests showed elevated liver enzymes (ALT 243 U/L, AST 234 U/L), prolonged PT (17s), and PTT (60s). Coagulation studies revealed deficiencies in protein C (18; normal 75-165), protein S (27; normal 50-120), and antithrombin III (38; normal 80-120). Factor V Leiden mutation was markedly elevated (290.4; normal >120), while ANA, anticardiolipin IgG, lupus anticoagulant, and other autoimmune and neoplastic markers were unremarkable. Ascitic fluid analysis was consistent with high-gradient, low-protein ascites (SAAG = 2.5).

The patient was initiated on anticoagulation therapy and diuretics. However, recurrent ascites and treatment noncompliance complicated management, leading to a poor prognosis. This case highlights the critical role of imaging, particularly Doppler ultrasound, contrast-enhanced CT, and MRI venography, in diagnosing BCS and emphasizes the need for early detection and evaluation of underlying prothrombotic conditions.

## Discussion

This case highlights a complex diagnostic challenge involving a 33-year-old female with a history of epilepsy and bipolar disorder, who presented with severe abdominal pain, ascites, and generalized abdominal distension. Her history of medication noncompliance, methamphetamine use, and psychiatric conditions further complicate the clinical picture.

Budd-Chiari syndrome (BCS) i3 characterized by hepatic venous outflow obstruction, which leads to liver congestion, hepatomegaly, and ascites. Acute BCS typically presents with sudden-onset abdominal pain, hepatomegaly, and ascites, with or without jaundice. The variability in BCS presentation often makes it the first manifestation of an underlying condition. For instance, BCS has been reported as a precursor to systemic lupus erythematosus (SLE), as seen in a case where a 32-year-old woman developed progressive abdominal distension over 4 months before being diagnosed with BCS, and subsequently SLE 6 months later[3,4]. Additionally, antiphospholipid syndrome (APS) has been identified as a primary cause of BCS, reinforcing the need to consider APS in differential diagnoses [5]. Emerging reports also associate BCS with COVID-19, where thromboembolic events may serve as the initial manifestation. One such case involved a 48-year-old woman diagnosed with BCS, who was later confirmed to have COVID-19, highlighting the need for vigilance regarding thrombotic complications associated with infections [6,7]. Furthermore, an unusual case reported the development of rhabdoid meningioma in a patient with a history of BCS, though the connection remains unclear, warranting further investigation [8]. These cases underscore the importance of recognizing BCS as a potential indicator of systemic disease.

In this case, the patient's symptoms—severe pain radiating to the right shoulder, nausea, and significant ascites—strongly suggested acute BCS. The ascitic fluid analysis showed a high serum-ascites albumin gradient (SAAG) of 2.4, consistent with portal hypertension secondary to BCS.

Imaging is crucial for diagnosing BCS, yet modalities such as Doppler ultrasound, CT, and MRI may not always yield definitive results. Doppler ultrasound can miss subtle cases, while CT and MRI require careful interpretation to differentiate BCS from other hepatic conditions. In this case, Doppler ultrasound demonstrated poor hepatic vein visualization, abnormal blood flow, and increased hepatic echogenicity. Subsequent CT imaging confirmed hepatomegaly, heterogeneous liver enhancement, hepatic vein narrowing, and prominent mesenteric veins—findings indicative of BCS. While liver biopsy can aid in diagnosis, it is not pathognomonic, and histological variability may add uncertainty, as highlighted by studies proposing histological scoring systems for BCS assessment [9]. The multifactorial nature of BCS, involving myeloproliferative disorders, malignancies, infections, and autoimmune diseases, further complicates its diagnosis, as illustrated in a case where BCS was the initial presentation of adult-onset Still's disease [10]. A multidisciplinary approach, including hepatologists, hematologists, and radiologists, is often necessary for timely diagnosis and optimal treatment.

The pathogenesis of BCS primarily involves a hypercoagulable state, which may be inherited (e.g., Factor V Leiden mutation, prothrombin gene mutation, protein C and S deficiencies) or acquired (e.g., APS, myeloproliferative disorders, pregnancy, infections such as COVID-19) [[11], [12], [13], [14], [15]]. For instance, COVID-19 has been linked to hepatic vein thrombosis due to its prothrombotic effects [11], while APS remains a well-established cause of BCS, demonstrating the strong link between autoimmune disorders and hypercoagulability [12]. Diagnosing BCS requires comprehensive laboratory workup and imaging to identify the underlying etiology and guide appropriate management, which typically includes anticoagulation therapy and targeted treatment for the hypercoagulable state [[11], [12], [13], [14], [15]].

Management of BCS follows a stepwise approach, beginning with anticoagulation for all patients to prevent thrombus propagation and facilitate recanalization [16]. Patients with short-segment hepatic vein or inferior vena cava obstruction may benefit from angioplasty or stenting [17], while transjugular intrahepatic portosystemic shunt (TIPS) is an effective intervention for relieving portal hypertension and improving liver function [16,18]. Surgical shunts are considered in refractory cases [16], and liver transplantation remains the definitive option for fulminant hepatic failure or unresponsive disease [16]. Prognosis is influenced by disease severity, underlying etiology, response to therapy, liver function, and complications such as hepatic encephalopathy [11,16,18,19]. Emerging research suggests that gut microbiome alterations and serum metabolomics may play a role in BCS prognosis, providing potential biomarkers for disease progression and therapeutic response [20,21].

**Fig. 1:**
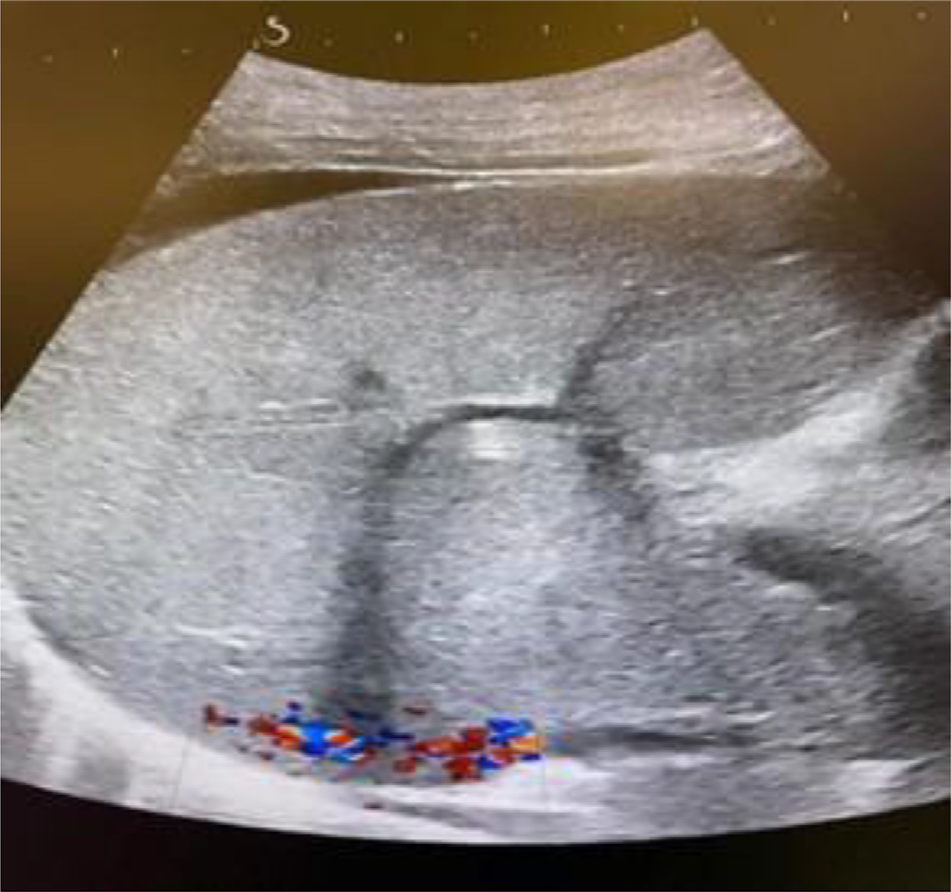
Ultrasound: Increased hepatic echogenicity. Poor visualization of hepatic veins. Abnormal Doppler flow, suggestive of hepatic vein obstruction

**Fig. 2:**
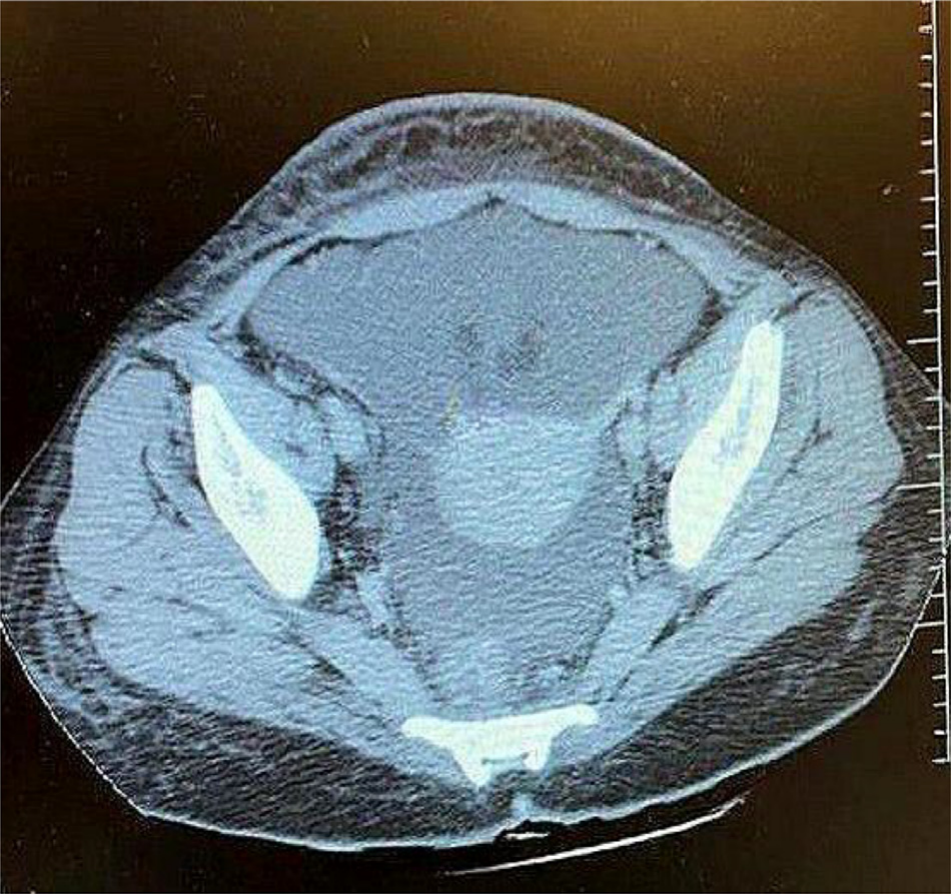
Pelvic CT scan: No significant pelvic mass or lymphadenopathy. Engorged pelvic veins, possibly due to venous congestion.

**Fig. 3:**
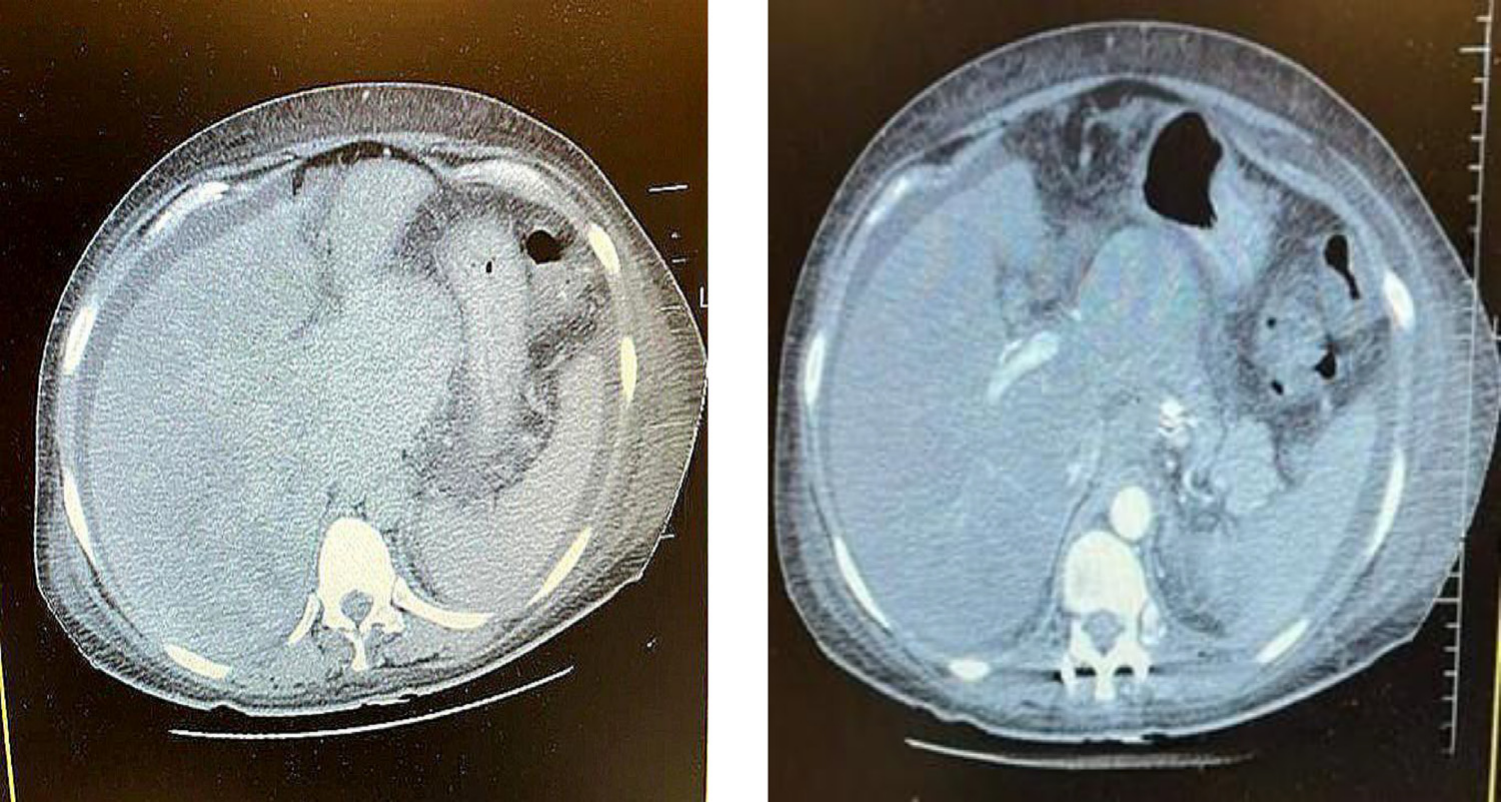
Abdominal CT scan: Hepatomegaly with heterogeneous enhancement. Narrowed or indistinct hepatic veins, supporting Budd-Chiari syndrome. Significant ascites and mesenteric venous congestion. Mild splenomegaly, suggesting portal hypertension.

In this case, the patient was treated with anticoagulation (warfarin) and diuretics (spironolactone, furosemide) per standard BCS management guidelines to prevent clot progression and control ascites. Despite initial stabilization, she experienced recurrent ascites, requiring multiple paracentesis procedures. This highlights the chronic and relapsing nature of BCS, particularly when underlying thrombotic risk factors remain unmanaged (Fig. 1, Fig. 2, Fig. 3).

Her history of methamphetamine use and aggressive behavior further complicated her clinical course. Methamphetamine is known to exacerbate hypercoagulable states, potentially worsening hepatic and systemic complications. The patient's sudden cardiac arrest raised suspicion of drug overdose, although the exact cause of death remains uncertain. Clinical suspicions included hyperkalemia, intracranial hemorrhage, and methamphetamine toxicity, but the absence of an autopsy prevents definitive conclusions.

This case underscores the diagnostic complexity of BCS, the importance of early recognition and anticoagulation therapy, and the impact of comorbid conditions and lifestyle factors on disease progression and outcomes**.**

## Conclusion

Budd-Chiari syndrome is a rare disorder characterized by hepatic venous outflow obstruction, often due to a hypercoagulable state. This case report emphasizes the importance of evaluating prothrombotic conditions in patients with BCS and the need for early diagnosis and timely treatment. A multidisciplinary approach involving hepatologists, hematologists, radiologists, and other specialists is often necessary for effective management. Understanding the underlying etiology of BCS is crucial for diagnosis and management, and regular follow-up and monitoring are essential for patients with this condition.

## Patient consent

Written informed consent was obtained from the patient for publication and any accompanying images. A copy of the written consent is available for review by the Editor-in-Chief of this journal on request

## References

[1] Iaquinta F., Sciacca E., Abatecola F., Fossati-Jimack L., Pitzalis C., Rivellese F. (2024). POS0351: a post-hoc analysis of the biopsy-driven, multicentre, randomised r4ra clinical trial reveals an innate immune signature in multi-drug resistant rheumatoid arthritis patients with a lympho-myeloid pathotype. Ann Rheum Dis.

[2] Gupta P., Bansal V., Kumar M.P., Sinha S.K., Samanta J., Mandavdhare H. (2020). Diagnostic accuracy of Doppler ultrasound, CT and MRI in Budd Chiari syndrome: systematic review and meta-analysis. Br J Radiol.

[3] Dremencov E., Lapshin M., Komelkova M., Tseilikman O., Tseilikman V. (2018). Role of dendritic spines in pathophysiology of depression. Gazzetta Medica Italiana Archivio per le Scienze Mediche.

[4] Solela G., Daba M. (2023). Budd-Chiari Syndrome as an initial presentation of systemic Lupus erythematosus associated with antiphospholipid syndrome: a case report with review of the literature. Open Access Rheumatol.

[5] Torres-Avelar H., Méndez-Nungaray D., Martínez-Núñez I. (2024). P4 Budd-Chiari syndrome as initial presentation of antiphospholipid syndrome: case report. Poster Presentat.

[6] Sh Hassan A.A., Alsaleh M.E., Alsaleh M.E., Al Zaher F.A., Almajed F.A., Alkhudhair A.M. (2021). Budd-Chiari syndrome: a case report of a rare presentation of COVID-19. Cureus.

[7] Sawaqed S.S., Urabi H.M., Al-Thnaibat M.H., Bani-Hani A., Mohd O.B., Mohd A.B. (2023). Budd-Chiari syndrome following COVID-19 infection: a case report. Ann Med Surg (Lond).

[8] Zeng Y., Zhang J., Jian W., Zhang Y., Yang Y., Li R. (2023). Rhabdoid meningioma with a history of Budd-Chiari syndrome: a case report and review of the literature. Front Oncol.

[9] Prasad P., Singh A., Singh A., Mishra P., Krishnani N. (2024). Significance of histopathological features in the diagnosis of Budd-Chiari syndrome on liver biopsies. Indian J Pathol Microbiol.

[10] Hakamifard A., Aria A., Momenzadeh M. (2023). Adult-onset still's disease and budd-chiari syndrome: a case report. Clin Case Rep.

[11] Gavriilidis P., Marangoni G., Ahmad J., Azoulay D. (2022). State of the art, current perspectives, and controversies of Budd-Chiari syndrome: a review. J Clin Med Res.

[12] Torun E.S., Erciyestepe M., Yalçınkaya Y., Gül A., İnanç M., Öcal L. (2022). A case of Budd-Chiari syndrome associated with antiphospholipid syndrome treated successfully by transjugular intrahepatic portosystemic shunt. Clin Med Insights Case Rep.

[13] Shimizu T., Yoshioka M., Ueda J., Kawashima M., Irie T., Kawano Y. (2024). Stenting of inferior right hepatic vein in a patient with Budd-Chiari syndrome: a case report. J Nippon Med Sch.

[14] Parikh P., Patel D., Shah R., Patel P. (2023). Unraveling the connection: PNH induced Budd-Chiari syndrome: a case study and early detection emphasis. Int J Sci Res (IJSR).

[15] Nikam V.G., Dhakre V.W., Motwani K., Chattopadhyay S. (2024). Budd-Chiari syndrome associated with congenital afibrinogenaemia reversed after orthotopic liver transplant. BMJ Case Rep.

[16] Hoekstra J., Janssen H.L. (2008). Vascular liver disorders (I): diagnosis, treatment and prognosis of Budd-Chiari syndrome. Neth J Med.

[17] Tripathi D., Sunderraj L., Vemala V., Mehrzad H., Zia Z., Mangat K. (2017). Long-term outcomes following percutaneous hepatic vein recanalization for Budd-Chiari syndrome. Liver Int.

[18] Shaker M.K., Sakr M.A., Dabbous H.M., Abdelhakam S.M., Samir A., Ebada H.A. (2018). Outcome of transjugular intrahepatic portosystemic shunt in Budd-Chiari syndrome: long-term outcomes of 118 patients: a single-center experience. Arab J Intervention Radiol.

[19] Wang Z.D., Ling S.B., Li S.X., Li L.H., Liu Z.C., Li D.Y. (2024). Analysis of risk factors of short-term prognosis in patients with severe Budd-Chiari syndrome. Zhonghua Wai Ke Za Zhi.

[20] Lu Q., Xu H., Zhou L., Zhang R., Li Z., Xu P. (2021). Alterations in faecal metagenomics and serum metabolomics indicate management strategies for patients with Budd-Chiari syndrome. Front Cell Infect Microbiol.

[21] Sun Y.L., Li W.Q., Ding P.X., Wang Z.W., Wei C.H., Ma X.X. (2018). Specific alterations in gut microbiota are associated with prognosis of Budd-Chiari syndrome. Oncotarget.

